# The Impact of Disease Severity on the Serum Levels of Significant Neutrophil Extracellular Trap (NET) Proteins in Patients with Psoriasis

**DOI:** 10.3390/ijms251910671

**Published:** 2024-10-03

**Authors:** Joanna Czerwińska, Agnieszka Owczarczyk-Saczonek

**Affiliations:** 1 Department of Dermatology, Sexually Transmitted Diseases and Clinical Immunology, The University of Warmia and Mazury, 10-719 Olsztyn, Poland; 2 Department and Clinic of Dermatology, Sexually Transmitted Diseases and Clinical Immunology; The University of Warmia and Mazury, 10-719 Olsztyn, Poland; agnieszka.owczarczyk@uwm.edu.pl

**Keywords:** psoriasis, neutrophils, neutrophil extracellular traps, NET, neutrophil elastase, myeloperoxidase, DNase I, citrullinated histones

## Abstract

Psoriasis is an inflammatory skin disease with various symptoms of differing severities and with the reported prominent involvement of neutrophil extracellular traps (NETs). The excitation of neutrophils, e.g., by interleukin 8 (IL-8) or lipopolysaccharide (LPS), leads to the citrullination of histones and the release of protein–DNA complexes into the extracellular space, where they are digested by DNases. Our aim was to explore data on the levels of protein-complexed DNAs neutrophil elastase–DNA (NE-DNA) and myeloperoxidase–DNA (MPO-DNA), citrullinated histones (citH2, citH3, citH4), and NET-degrading enzyme DNase I in the serum of psoriatic patients with varying severities of clinical symptoms assessed with the Psoriasis Area Severity Index (PASI), Body Surface Area (BSA), and Dermatology Life Quality Index (DLQI) scores. The levels of factors were detected in 52 patients with psoriasis and 22 healthy volunteers by the enzyme-linked immunosorbent assay (ELISA). The results showed the elevated levels of NE-DNA, MPO-DNA, citH3, and DNase I in the patients with psoriasis compared to healthy volunteers (*p* < 0.05). Additionally, changes were noticed in the levels of NE-DNA, citH3, and DNase I, depending on the severity of symptoms (*p* < 0.05). In mild psoriasis (PASI < 10, BSA < 10, DLQI < 10), the suppressing activity of the enzyme caused the impaired ability to remove the physiological level of NETs, whereas in moderate to severe psoriasis (PASI ≥ 10, BSA ≥ 10, DLQI ≥ 10), the enhanced activity of DNase I failed to remove NETs due to the observed overexpression. It may, thus, be concluded that the mechanism of action of NETs, which play an undeniable role in psoriatic diseases, seem to follow two different paths depending on the severity of disease, which may be crucial in selecting potential anti-NET treatment methods.

## 1. Introduction

Psoriasis is an inflammatory skin disease with the prominent involvement of short-lived cells neutrophils, not only appearing as a skin infiltration, but also being manifested by the production of the long-lasting neutrophil extracellular traps (NETs). The NETs, abundant in the cytoplasmic granules and DNA, strongly affect the well-defined interaction in psoriasis, namely the control of the interleukin tumor necrosis factor α/interleukin—17/interleukin-23 (TNFα/IL-17/IL-23) cytokine axis by interacting with lymphocytes, dendritic cells, and keratinocytes [1,2,3,4]. NET formation starts from neutrophil activation by both intracellular and extracellular microorganisms (bacteria, fungi, parasites), i.e., by lipopolysaccharides (LPS) produced by most Gram-negative bacteria (very often used as direct stimuli factor for NET creation in in vitro study) as well as antibodies and immune complexes, chemokines, and cytokines, i.e., interleukin 8 (IL-8), interferon (IFN), complement factor 5a, TNFα, phorbol-12-myristate-13-acetate (PMA), monosodium urate crystals, and nitric oxide [4,5]. This activation results in the stimulation of protein arginine deiminase 4 (PAD-4, a Ca2+-dependent enzyme), which is responsible for chromatin decondensation through histone citrullination (citH2, citH3, citH4) [6]. The last stage entails the release of proteins, e.g., neutrophil elastase (NE) and myeloperoxidase (MPO)—triggering histone degradation and modifying the chromatin structure, from neutrophil granules [7], citrullinated histones, as well as a modified DNA chain from the nucleus in the form of complexes, e.g., NE-DNA or MPO-DNA, into the extracellular space as NETs [1,2,3,4].

Undoubtedly, the physiological role of NETs is very important but, on the other hand, the large amounts of DNA, hormones, and proteins stored in cells or released outside, observed in pathological conditions, are increasingly presented as the cause of the development of immune-mediated diseases, including psoriasis. The development of other autoimmune diseases, such as systemic lupus erythematosus (SLE) or rheumatoid arthritis (RA) entailing the overexpression of NETs (an increase in the concentrations of the NET proteins), is also triggered by the production of autoantibodies against NET components, including double-stranded DNA (dsDNA), nucleosomes (NUC), and histones (HI) in SLE; neutrophil cytoplasm (NC) in vesicular diseases; and cyclic citrullinated peptide (CCP) in RA. Studies addressing NETs in SLE have also focused on the involvement of low-density granulocytes (LDGs), which showed the enhanced production of inflammatory cytokines, causing the increased production of antigens, as well as oxidized mitochondrial DNA and thus mitochondrial ROS, making them more potent immunostimulants [8]. In turn, the development of RA correlated with both the accumulation of DNA-MPO as well as antibodies against citrullinated histones [9]. Additionally, the overexpression of NETs makes the removal of their residues by DNases (e.g., DNase I) insufficient. Thus, it may aggravate inflammation, disrupt microcirculation, and cause tissue damage [10].

Analyses of the NET production in various disease entities allow the development of two main therapeutic approaches: (1) drugs preventing both the accumulation and activation of neutrophils as well as the formation of NETs by inhibiting the production of cytokines (e.g., IL-8) and using inhibitors of components involved in NET activation, i.e., NE; and (2) drugs destroying already existing NETs using DNase I and antioxidants targeted at mitochondria to inhibit ROS formation [8].

In recent years, the involvement of NETs in the development of autoimmune diseases has been broadly addressed in scientific research. Previous investigations assessing the activity of NETs (directly or indirectly by measuring their products) were performed in patients with SLE, lupus nephritis, psoriatic arthritis, rheumatoid arthritis, as well as psoriasis [9,11,12,13,14]. The conclusions drawn from these studies were generally compliant and demonstrated the enhanced NET activity presented as NETotic cells (the ratio of neutrophils with stained extracellular DNA to the total number of neutrophils) and/or unbound NE, MPO in peripheral blood, serum, and/or the lesional skin of patients [9,11,12,13,14]. So far, only one study has reported the correlation between NETotic cells in peripheral blood and psoriasis severity [3]. Despite that, the complete understanding of the involvement of NETs in the psoriasis-induced inflammation still poses a challenge. There is a lack of comprehensive data about circulating markers of NETs and the correlations between them in patients at different stages of diseases, which could be useful in developing potential anti-NETs therapeutics. Our aim was to fill the gap in the data on the levels of the protein-conjugated DNA (the products of neutrophils with high objectivity and specificity to NETs), citrullinated histones, and a NET-degrading enzyme in the serum of psoriatic patients with varying severities of clinical symptoms.

## 2. Results

First, blood samples of all study participants (patients and controls) were analyzed for the levels of neutrophils and C Reactive Proteins (CRPs). The level of neutrophils was the highest in patients with mild psoriasis (56.9 ± 2.19 and 3.8 ± 0.41, *p* < 0.05), whereas the CRP concentration was elevated in patients with the moderate/severe course of the disease, but the values of both variables were within normal ranges (Table 1).

Next, the concentrations of the main NET stimuli factors lipopolysaccharide (LPS) and interleukin-8 (IL-8); NET markers neutrophil elastase–DNA (NE-DNA), myeloperoxidase–DNA (MPO-DNA); citrullinated histones H2, H3, and H4 (citH2, citH3, citH4); and DNAse I, the enzyme responsible for the degradation of NETs, as well as the correlations between these proteins, were analyzed in the serum samples of the psoriatic patients with different severity forms of the disease.

**Figure 1:**
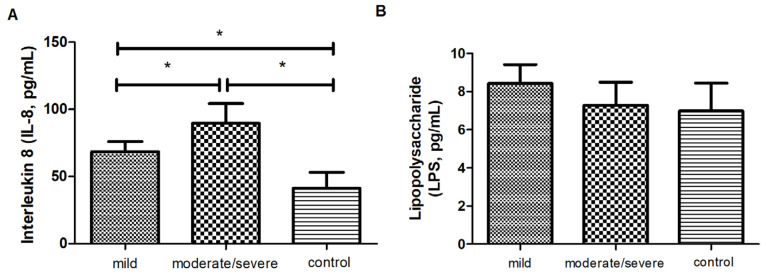
The mean levels of (**A**) interleukin 8 (IL-8, pg/mL) and (**B**) lipopolysaccharide (LPS, pg/mL) in the blood serum of the patients with different symptoms of psoriasis (mild, *n* = 19; moderate/severe, *n* = 39) and in the control group (*n* = 22). * indicates a statistically significant difference (*p* < 0.05).

Among the stimuli factors of NETs, only the level of IL-8 was changed and higher in the psoriatic patients than in the healthy controls (*p* < 0.05), with the highest value noted in the group with PASI ≥ 10 (89.7 ± 7.59 pg/mL, *p* < 0.05, Figure 1A). The LPS concentration did not differ significantly between the patients and healthy volunteers (8.4 ± 0.99 pg/mL ver. 7.3 ± 1.19 pg/mL ver. 6.9 ± 1.46 pg/mL, *p* = 0.16) (Figure 1B).

**Figure 2:**
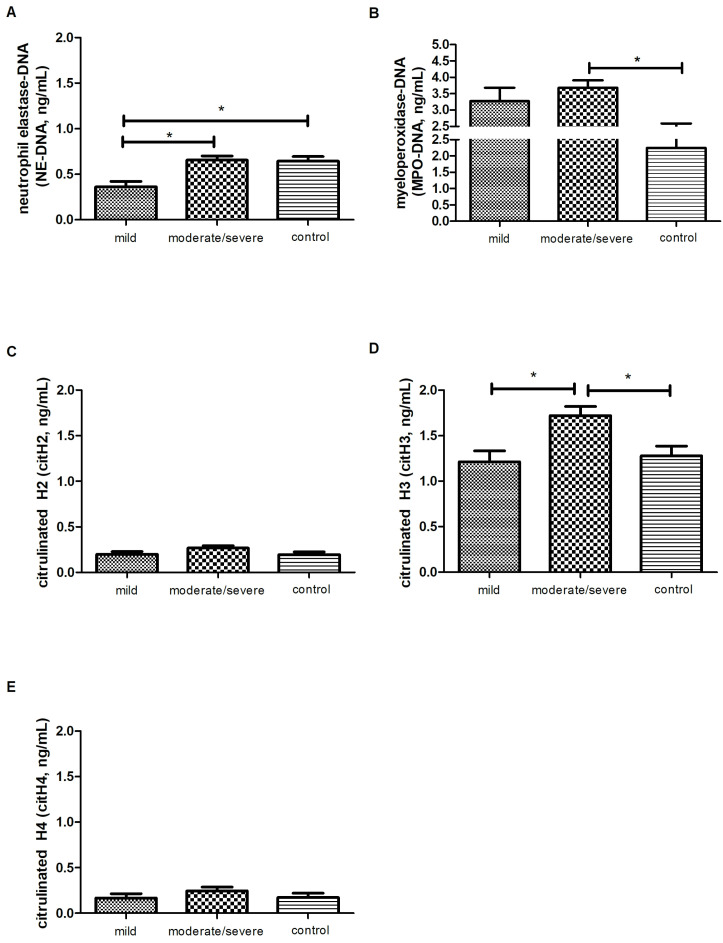
The mean level of (**A**) neutrophil elastase–DNA complex (NE-DNA, ng/mL), (**B**) myeloperoxidase–DNA complex (MPO-DNA, ng/mL), (**C**) citrullinated H2 (citH2, ng/mL), (**D**) citrullinated H3 (citH3, ng/mL), and (**E**) citrullinated H4 (citH4, ng/mL) in the blood serum of the patients with different symptoms of psoriasis (mild, *n* = 19; moderate/severe, *n* = 39) and in the control group (*n* = 22). * indicates a statistically significant difference (*p* < 0.05).

In the group of proteins considered as the markers of NET formation, significant differences were noted in the levels of NE-DNA, MPO-DNA, and citH3. The lowest concentration of the NE-DNA complex was determined in the group of patients with PASI < 10 (0.2 ± 0.04 ng/mL, *p* < 0.05, Figure 2A). The level of MPO-complexed DNA was elevated in the psoriatic patients with moderate or severe symptoms compared to normal controls (3.9 ± 0.24 ng/mL ver. 2.2 ± 0.34 ng/mL, *p* < 0.05, Figure 2C). The concentration of citrullinated histones citH2 and citH4 was stable in all study groups (*p* = 0.1088 and *p* = 0.3660, respectively; Figure 2C,E), whereas the level of citH3 was higher (*p* < 0.05) in patients with Psoriasis Area Severity Index (PASI) scores ≥10 (1.7 ± 0.10 ng/mL) compared to the group with PASI < 10 (1.2 ± 0.12 ng/mL) and healthy volunteers (1.3 ± 0.11 ng/mL) (Figure 2D).

**Figure 3:**
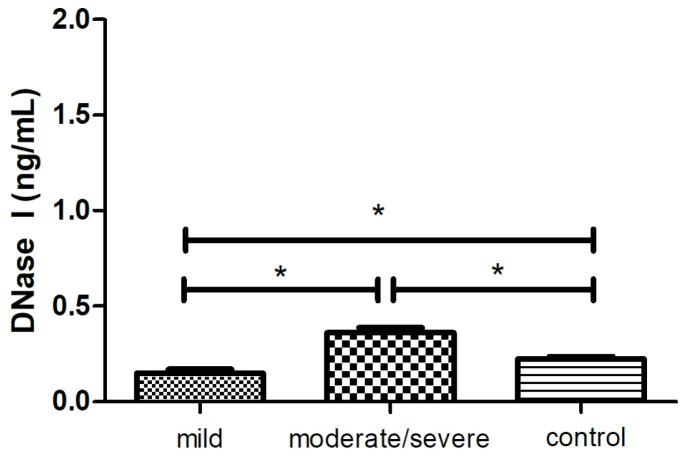
The mean level of DNase I (ng/mL) in the blood serum of the patients with different symptoms of psoriasis (mild, *n* = 19; moderate/severe, *n* = 39) and in the control group (*n* = 22). * indicates a statistically significant difference (*p* < 0.05).

The analysis of the DNase I concentration showed lower values (0.1 ± 0.02 ng/mL, *p* < 0.05) in the patients with a mild form of psoriasis (PASI < 10) and higher levels (0.3 ± 0.02 ng/mL, *p* < 0.05) in the group with a moderate/severe course of psoriasis compared with the healthy controls (0.2 ± 0.01 ng/mL, *p* < 0.05, Figure 3).

The analysis of correlations between all analyzed factors indicated a significant correlation between the level of IL-8, the NE-DNA complex, citH3, and DNase I and the severity of psoriasis expressed as PASI, Body Surface Area (BSA), and Dermatology Life Quality Index (DLQI) scores (*p* < 0.05, Table 2).

In the group of patients with mild psoriasis, there were positive correlations between DNase I and NE-DNA (r = 0.6016, *p* < 0.05), MPO-DNA and NE-DNA (r = 0.5297, *p* < 0.05), as well as citH3 and MPO-DNA (r = 0.5679, *p* < 0.05). In turn, in the group of moderate/severe psoriatic patients, a negative correlation was found between the NE-DNA complex and citH3 (r = −5659, *p* < 0.05).

## 3. Discussion

The current study has, for the first time, demonstrated the presence and fluctuations of significant NET proteins and complexes (remnants), as well as their main digestive enzyme in the serum of psoriatic patients, and the correlation of all factors studied with the stage of the disease. In the study group, the levels of neutrophils and CRP fell within the normal ranges. In turn, the levels of NE-DNA, MPO-DNA, citH3, and DNase I determined in the psoriatic patients differed from those assayed in the healthy volunteers. Additional changes were noticed in the levels of NE-DNA, citH3, and DNase I, depending on the severity of symptoms. These dependences were confirmed by correlations with the PASI, BSA, and DLQI scores. The distribution and physiological role of NETs have been extensively discussed in scientific research; however, the explanation of NET implication in psoriasis development is still problematic.

Among the stimuli factors of NETs, the level of IL-8 was higher in the patients compared to healthy controls, with the highest level determined in the group with PASI ≥ 10. Its values were also correlated with the PASI, BSA, and DLQI scores. This phenomenon was easy to predict due to the fact that ample publications have already confirmed the role of IL-8 as a marker of the severity of psoriasis [15]. It was also a confirmation that the study groups were created properly (positive control). IL-8 is a well-known pro-inflammatory and growth-promoting factor that induces keratinocyte overgrowth in psoriasis. In the available literature, we found studies analyzing both IL-8 serum levels and tissue protein expression. IL-8 levels in patients were significantly higher than in the control group [16] and correlated with the disease stage, which indicates that they are sensitive biomarkers for assessing the efficacy of psoriasis therapy [17]. IL-8 concentrations changed significantly over the treatment and are correlated with skin condition improvements. Immunohistochemistry staining showed that IL-8 was the only cytokine expressed in the affected skin of all patients, but not in the clinically normal skin of healthy individuals. In seven CD4+ T cell clones expanded from the affected skin of an untreated patient with psoriasis, IL-8 was expressed by skin-derived T cells. IL-8 mRNA was not detected in the skin of any patient after the initiation of a systemic therapy [18]. Many studies have shown IL-8 overexpression in the skin to be associated with the accumulation of large amounts of neutrophils, while neutrophil infiltration relates to inflammatory and treatment-resistant psoriasis. IL-8 was positively stained only in neutrophils, but not in macrophages or keratinocytes, which suggests that neutrophils are one of the sources of IL-8 in psoriasis. Furthermore, the stimulation of human neutrophils with the LPS or the tumor necrosis factor-alpha (TNFα) induced IL-8 production in vitro [19]. Additionally, other research has shown the distinct localization of IL-8 and complement components, suggesting that the intraepidermal migration of neutrophils first follows the IL-8 concentration gradient, and then they are guided by complement components to their final destination, namely the subcorneal part of the diseased skin [20].

Surprisingly, the LPS concentration was stable in each study group. The results were, however, in opposition to studies showing the increased LPS level in the blood serum of psoriatic patients [21] and the suppressed proliferation of HaCaT in mice cells after decreasing serum LPS levels [22]. Additionally, a clear anti-inflammatory effect was observed through the inhibition of the p65 NF-κB signaling cascade induced by LPS/imiquimod and the strong inhibition of the cytokine storm production associated with psoriasis-like skin inflammation, which led to the restoration of normal epidermal structure [23].

Discussing the results of MPO-DNA and NE-DNA was problematic because we found only one original article in the available literature where the level of MPO-conjugated DNA was analyzed in psoriatic patients [9]. In our study, the level of MPO-DNA was markedly elevated in the moderate/severe patients, but was not correlated with the severity of the disease. Similar results were obtained by Li et al. (2022) who reported that the serum level of the MPO-DNA complex was significantly increased in patients with both psoriatic arthritis (PsA) and psoriasis compared to healthy controls. Moreover, the baseline MPO-DNA level and change in MPO-DNA during treatment were associated with skin condition improvement in the case of patients with PsA, but not with the PASI score in psoriasis. Thus, the cited authors suggested that serum MPO–DNA may be used as a biomarker to predict the therapeutic response in patients with PsA [9]. An increased serum level of the MPO–DNA complex has also been found in other autoimmune diseases, such as rheumatoid arthritis, Behcet’s disease, and ANCA-associated vasculitis [24,25,26,27,28], but still without any dependence on skin condition. Other studies have demonstrated changes in the serum or tissue level of free/unbound MPO. Here, the results of Cretu et al. (2018) confirmed the previous cited reports and showed that not only MPO-DNA but also unbound MPO levels may be used as markers for PsA [29]. The elevated levels of MPO (in serum and skin) were also noted in psoriatic patients [30,31]. In the study by Cao et al. (2014), the serum level of MPO was elevated in psoriatic patients, but, again, without any correlation with psoriasis severity. MPO was also highly expressed in lesional psoriatic skin and was colocalized predominantly with CD45(+) CD11b(+) leukocytes. The CD11b(+) cell density correlated with the circulation MPO levels. Thus, MPO seems to be a biomarker for systemic inflammation related to cardiovascular disease [32]. The elevated concentration of MPO in the samples taken from both healthy subjects as well as psoriatic patients after LPS treatment was determined in the study by Wójcik et al. (2020) [33]. In contrary, no significant difference in MPO expression was found between healthy and psoriatic groups in the study by Kirmit et al. (2020) [34].

In the case of MPO, much attention is focused on genetic research. The role of the gene encoding MPO is especially discussed in the development of generalized pustular psoriasis. Mutations in the MPO gene affected changes in neutrophil activity, whereas MPO deficiency caused defective NET formation and reduced the phagocytosis of neutrophils by monocytes. Additionally, it was noted that the activity of MPO was inversely correlated with NE and proteinase 3, which regulate the IL-36 pathway [35,36].

Interestingly, in our study, a positive correlation was also found between MPO-DNA and NE-DNA, but only in the group of psoriatic patients with a mild form of psoriasis. Additionally, in the present study, the concentration of NE-complexed DNA was considerably lower in the patients with mild psoriasis and higher in those with moderate or severe symptoms compared to the healthy controls. These observations were also confirmed by positive correlations with the PASI, BSA, and DLQI. In the present study with psoriatic patients (without division on the stage of psoriasis), the concentration of NE was higher in the neutrophils and blood plasma of the patients compared to the controls. However, unlike MPO, its level did not differ after LPS treatment [33]. The high expression of NE was also observed in the epidermis of lesional skin in psoriasis [3].

In the studies addressing the pathogenesis of psoriasis, more and more attention is paid to the process of histone citrullination in neutrophils, which initiates NETs. Although histones are conservative and weak immunogens, citrullination clearly increases their immunogenicity and triggers the production of autoantibodies [37]. Th excessive activation of the PAD-4 enzyme, which initiates histone citrullination, is observed in neutrophils. Studies into PAD-4 protein expression in the blood serum of patients with psoriasis have shown its increased level compared to the control group before the treatment and a decrease in the enzyme concentration as a result of systemic therapies [38]. Concerning the pathogenesis of psoriasis, attention is also paid to the physiological citrullination of keratin proteins and filaggrin, which allows for the preservation of the barrier function of the epidermis [39]. Here, in turn, it was the deficiency of citrullination in the epidermis, caused by the deficiency of the PAD-1 enzyme, that caused extreme keratinization and inflammatory reactions [40]. In recent decades, a number of studies have indicated the dysregulation of the citrullination of extracellular proteins as a factor driving the autoimmune response, e.g., in RA [41]. Among citrullinated proteins, there are also histones, e.g., the most well-known being H3. The enhanced citrullination of H3 was observed in CD14hi monocytes, as well as CD1c+ dendritic cells and CD66+ granulocytes. The number of CD14hi monocytes with enhanced H3 citrullination was significantly increased in the individuals with positive antibodies recognizing citrullinated proteins (anti-cyclic citrullinated peptide (anti-CCP)-positive, ACPA+) [42]. An increased level of antibodies against citH3, released into the extracellular space, has also been demonstrated in the patients with a severe form of RA—Palty’s syndrome. The involvement of citH3 in the development of the disease has also been confirmed in the studies conducted on a mouse model, where a decrease in the expression of the gene encoding PAD-4 was associated with a reduction in the severity of the RA course [43].

Our observation of citrullinated histones indicated that only the serum level of citH3 differed between the stages of psoriasis, with its highest value being noted in the group of patients with moderately/severely severe psoriasis. Additionally, different correlations were found depending on the disease symptoms, i.e., in the group of patients with mild psoriasis, a positive correlation was reported between citH3 and MPO-DNA, whereas in the group of moderate and severe psoriasis, there was a negative correlation between the NE-DNA and citH3. Similar findings were obtained in a study of RA, where the elevated serum level of citH3 was correlated with the levels of cell-free DNA (cfDNA) [44]. Although we did not find any studies performed in the serum of patients diagnosed with psoriasis, the expression of histones was analyzed in psoriatic skin, where the H2A was strongly presented in the epidermis [3].

Currently, studies of histones in psoriasis also entail their post-translational modifications, mainly the methylation and acetylation of histone H3 (K27 and K4), which play an important role in the regulation of cytokine production and the response to biological drugs taken by patients with psoriasis [45,46,47,48]. Interestingly, a comparison of histone H3 acetylation between psoriatic and healthy skin showed its overexpression in the diseased tissue [49,50]. In turn, reduced levels of acetylation in H3 and H4 were determined in peripheral blood mononuclear cells, and additionally, the level of H4 acetylation was found to negatively correlate with the severity of the disease [51,52].

As it turns out, not only the formation of the NETs, but also their removal by DNases play a significant role in the activity of the autoimmune diseases. Our findings provided novel information about the dependence of DNase I level on the severity of psoriasis. The decreased concentration of DNase I was noted in the group of patients with mild symptoms of psoriasis (positive correlation with NE-DNA), whereas its level in moderate/severe psoriasis was increased compared to the healthy volunteers.

The role of DNase I in NET degradation has been widely studied, especially in SLE [8,53,54], where it was shown, a.o., that the ability to degrade NETs changed with disease activity, achieving the highest values during remission. The overall impaired endonuclease activity has been associated with (1) an increased presence of DNase I inhibitors; (2) an increased concentration of anti-NET antibodies (including anti-DNA), which prevent DNase I from accessing NETs [53,54]; (3) the mutation of the gene encoding DNase I [55]; and (4) a positive feedback loop, where C1q deposited in NETs acts as a DNase I inhibitor and additionally promotes the production of antibodies [56].

Additionally, DNase was able to remove only DNA from NETs, but not the proteins attached. This is due to the fact that NET residues bind secondarily to the host vascular endothelium [57], causing it to damage its own tissues and triggering the development of diseases [7]. These reports were confirmed by studies examining DNase I activity in psoriasis and SLE. In psoriasis, the dysregulation of DNase I in CD14+ cells associated with the incomplete degradation of the DNA in the epidermis that caused parakeratosis (49, 58) has been assumed to play a role in the etiology of parakeratosis through the incomplete degradation of DNA in the epidermis [58,59]. There was, however, no information about the dependence of DNase I levels on the severity of symptoms in psoriasis. However, in SLE and CLE patients, the DNase I concentration was significantly lower than in healthy controls [60,61], and in SLE, it was positively correlated with disease severity [61]. In the experiment with the propylthiouracil (PTU)-induced lupus-like syndrome, patients had lower DNase I activity than the SLE patients and controls. DNase I activity was increased after the discontinuation of PTU, but its level was still lower than in the controls [62].

Based on the above data and the results obtained, we can conclude that NETs are not stabilized by autoantibodies in psoriasis, and thereby, they do not affect DNase I activity. Thus, DNase I expression is not inhibited in psoriasis as intensively as in other autoimmune diseases, including SLE. Therefore, the reduced level of DNase I, observed in the group of patients with mild psoriasis, may be related to the activity of inhibitors and/or insufficient stimulation by NETs (reduced levels of NE-DNA and physiological levels of MPO-DNA and citrullinated histones). However, as the patients’ symptoms were mild, DNase I was partially functional despite the reduced levels, whereas in the group of severe patients, DNase I was ineffective despite its increased concentration, which led us to believe that it was most likely blocked by inhibitors. The obtained results concerning the role of DNase I itself in NETs degradation indicate the validity of work on targeted therapies, i.e., various treatment options for patients depending on the severity of symptoms—the administration of exogenous DNase I in mild psoriasis (promising results in in vitro experiments of [63]) or minimizing the activity of enzyme inhibitors in severe psoriasis. The paucity of data on DNase I inhibitors in psoriasis makes this area ripe for further study.

## 4. Materials and Methods

### 4.1. Study Group

The study group included 57 psoriatic patients (Table 3) treated in the Clinic and at the Department of Dermatology, Sexually Transmitted Diseases and Clinical Immunology in Olsztyn (Poland). Patients with other inflammatory diseases—neoplastic diseases; previous cardiovascular complications; as well as heart, kidney, and liver failure—were excluded. The control group consisted of healthy volunteers (*n* = 22) with no personal and family history of inflammatory and autoimmune diseases. The study was approved by the Bioethical Committee of the Warmia and Mazury University in Olsztyn (Poland) (Resolution 60/2019 and 1/2013). Informed consent was obtained from each patient enrolled in the study. Patients were classified into two research groups depending on skin condition, as assessed by the PASI, BSA, and DLQI. The first group consisted of patients (*n* = 19) with a mild form of psoriasis (PASI < 10, BSA <10, DLQI <10), whereas the second one (*n* = 39) included patients with more moderate/severe phases of the disease with PASI ≥10, BSA ≥10, and DLQI ≥ 10. All patients underwent systemic treatment of psoriasis for at least 4 weeks or topical treatment for 1 week prior to enrolment in the study. We also selected patients whose systemic therapy (methotrexate, anti-IL17, or anti-TNFα) was the shortest (discontinued due to treatment ineffectiveness; patients were prepared to the next therapy). In mild psoriasis, some patients used a topical drug containing Calcipotriol/Betamethasone Dipropionate. However, in both groups, the majority of patients were newly diagnosed, without any general or local therapies in the history. Table 4 presents mean values of disease markers.

### 4.2. Methods

Participants from both study groups had their blood samples subjected to hematological and biochemical analyses of blood, including levels of neutrophils and C Reactive Protein (CRP), that were performed in the Laboratory of the Municipal Polyclinical Hospital in Olsztyn. The LPS, IL-8, citH2, citH3, citH4, NE-DNA, MPO-DNA, as well as DNase I levels in blood serum were assessed by the enzyme-linked immunosorbent assay (ELISA). Commercially available kits (Enlibio, Wuhan, China; Cusabio, Wuhan, China) with a wide detection range were used as follows: 6.25–400 pg/mL for LPS (CSB-E09945h); 15.625–2000 pg/mL for IL-8 (CSB-E04641h); 0.312–20 ng/mL for citH2 (EIA08443h), citH3 (EIA08444h), citH4 (EIA08445h), NE-DNA (EIA09793h), and MPO-DNA (EIA09433h); and 0.156–10 ng/mL for DNase I (EIA05573h). The analytical procedures were performed according to the manufacturer’s protocol. The assay’s validity was confirmed based on parallelism between the standard curve and dilutions of randomly chosen serum samples. The intra- and inter-assay coefficient of variation for factors was <8% and <12%, respectively. The sensitivity of these assays was defined as the least concentration that could be differentiated from zero samples, and it was determined at 1.56 pg/mL (LPS), 7 pg/mL (IL-8), 0.06 ng/mL (citH2, citH3, citH4, NE-DNA, and MPO-DNA), and 0.05 ng/mL (DNase I). Absorbance values were measured at 450 nm using Multiskan FC (Thermo Fisher Scientific, Waltham, MA, USA).

### 4.3. Statistical Analysis

After the assumptions of the normality and homogeneity of variances had been checked, the results were processed statistically with non-parametric tests (the system was not in accordance with normal distribution). An ANOVA Kruskal–Wallis test was used to compare each of the studied group with the control, and Spearman’s test was performed to assess the correlation between factors. All statistical analyses were performed using Statistica software (ver. 13, StatSoft Inc., Tulsa, OK, USA). Differences were considered statistically significant at *p* < 0.05. The results were expressed as means ± SEM.

## 5. Conclusions

The study confirmed the involvement of NETs in psoriasis pathogenesis. The levels of the analyzed factors—NE-DNA, citH3, and DNase I—varied under the influence of the severity of the disease. Interestingly, NETs, which play an undeniable role in psoriatic diseases, seem to act in two different ways depending on disease severity. A decrease in the NE-DNA level and a positive correlation with the DNase I level were observed in mild psoriasis. The activity of the enzyme might be impaired or blocked, and its low activity was not able to remove the resulting NET, the levels of which were within the physiological range and comparable with the control level of MPO-conjugated DNA and citH3. In turn, a significantly increased level of DNAse I together with evaluated concentrations of citH3 and the MPO-DNA complex were noted in moderate and severe psoriasis. It may be suggested that despite the higher activity of DNase I, the NETs could not be removed due to their overexpression (a high level of citH3 and MPO-DNA with a physiological level of NE-DNA). The presented data may prove important in the development or selection of potential anti-NET treatment methods for patients with different courses of psoriasis. To evaluate the mechanism of action of NET compounds, it is important to perform more studies also in lesional and non-lesional skin.

## Figures and Tables

**Table 1:** Laboratory analysis of blood serum samples of patients with mild psoriasis (*n* = 18), moderate/severe psoriasis (*n* = 39), and controls (*n* = 22); CRP—C Reactive Protein; ↑* indicates a statistically significant difference (*p* < 0.05).

	Neutrophils (%)	Neutrophils (10^/uL)	CRP (mg/L)
Range	40–70	1.8–7.7	<5
Mild psoriasis (*n* = 18)	56.9 ± 2.19↑ *	3.7 ± 0.41↑ *	1.0 ± 0.19
Moderate/severe psoriasis (*n* = 39)	50.9 ± 1.76	3.2 ± 0.27	3.0 ± 0.73↑ *
Control group (*n* = 22)	51.5 ± 1.72	2.7 ± 0.19	0.8 ± 0.07

**Table 2:** The correlation coefficient (r) between interleukin-8 (IL-8), neutrophil elastase (NE-DNA), citrullinated histone H3 (citH3), and DNase I and the Psoriasis Area Severity Index (PASI), Body Surface Area (BSA), and Dermatology Life Quality Index (DLQI) scores in the psoriatic patients (*n* = 57, *p* < 0.05).

	IL-8	NE-DNA	citH3	DNase I
PASI	0.3874	0.5625	0.3151	0.4950
BSA	0.3784	0.5503	0.3503	0.5015
DLQI	0.2781	0.6096	0.2910	0.4925

**Table 3:** Clinical characteristics of patients with psoriasis.

	All Participants	Mild Psoriasis PASI < 10, BSA < 10, DLQI < 10	Moderate/Severe Psoriasis PASI ≥ 10, BSA ≥ 10, DLQI ≥ 10	Healthy Volunteers
Numbers (*n*)	*n* = 57	*n* = 18	*n* = 39	*n* = 22
Males/Females (M/F)	36 M/21 F	8 M/10 F	28 M/11 F	10 M/12 F
Age (years)	45.8 ± 1.89	38.5 ± 3.10	48.9 ± 2.18	36.1 ± 1.92

**Table 4:** The mean levels of PASI, BSA, and DLQI markers in the group of patients with mild (*n* = 18) and moderate/severe (*n* = 39) psoriasis.

	Mean Value		
	PASI	BSA	DLQI
Mild psoriasis (*n* = 18)	3.0 ± 0.43	3.5 ± 0.71	5.3 ± 1.21
Moderate/severe psoriasis (*n* = 39)	14.3 ± 0.74	25.7 ± 2.74	17.4 ± 0.86

## Data Availability

Data are contained within the article.
